# A survival model for prognostic prediction based on ferroptosis-associated genes and the association with immune infiltration in lung squamous cell carcinoma

**DOI:** 10.1371/journal.pone.0282888

**Published:** 2023-03-16

**Authors:** Yanyi Lu, Hua Yang, Yunliang Cao, Yunan Wang, Mengjia Wu, Bo He, Junzhu Xu, Zixuan Su, Wen Luo, Yuyang Liu, Wei Hu

**Affiliations:** 1 Department of Thoracic Oncology, The Second Affiliated Hospital of Zunyi Medical University, Zunyi City, Guizhou Province, China; 2 Department of pathology, The Second Affiliated Hospital of Zunyi Medical University, Zunyi City, Guizhou Province, China; University of Nebraska Medical Center, UNITED STATES

## Abstract

Lung squamous cell carcinoma (LUSC) is the primary pathological type of lung cancer with a less favorable prognosis. This study attempts to construct a ferroptosis-associated signature associated with overall survival (OS) that can predict the prognosis of LUSC and explore its relationship with immune infiltration. A 5 ferroptosis-associated gene model was constructed by LASSO-penalized regression analysis to predict the prognosis of patients with LUSC in the TCGA database and validated in the GEO and TCGA databases. Patients were stratified into high-risk and low-risk groups by the median value of the risk scores, and the former prognosis was significantly worse (P<0.001). Additionally, we found a certain association between the two risk groups and immune infiltration through CIBERSORT. Meanwhile, the differentially expressed genes (DEGs) between normal and tumor tissue were used to perform functional analysis, which showed a significant association with leukocyte transendothelial migration pathways in the TCGA cohort. In addition, immune cell infiltration analysis confirmed that M2 macrophages were significantly highly expressed in the high-risk group. Overall, the model successfully established by ferroptosis-associated genes suggests that ferroptosis may be related to immune infiltration in LUSC.

## 1. Introduction

With an estimated 2.2 million new cases and 1.8 million deaths, lung cancer was the second most commonly diagnosed cancer and the leading cause of cancer death in 2020 [1]. According to histological types, lung cancers are mainly classified into small cell lung cancer (SCLC) and non-small cell lung cancer (NSCLC). NSCLC represents 87% of all lung cancers, and approximately one-third of NSCLC cases are reported as LUSC [2]. LUSC is associated with poor clinical prognosis and lacks targeted agents or other treatments compared to lung adenocarcinoma, although immunotherapy comes into play [3, 4]. The reasons for the dismal prognosis are the absence of valid therapeutic targets and a shortage of effective prognostic biomarkers for guidance on cancer therapy. Therefore, it is essential to identify a prognostic model, and further research on this basis is needed to identify possible factors affecting the prognosis to provide treatment options and improve the prognosis in patients with LUSC. The rapid development of bioinformatics analysis has enabled access to gene expression and clinical information through different databases, such as TCGA, GEO and ICGC. These resources provide a novel way to establish a prognostic prediction model to evaluate prognosis in LUSC, which can also be used to provide more ideas and directions for in-depth internal mechanism research and then guide clinical practice.

Ferroptosis was recently identified as being different from apoptosis and other primary forms of regulated cell death (RCD) in many aspects, and it was formally designated as a new method of cell death by Brent R. Stockwell’s laboratory in 2012 [5]. Morphologically, ferroptosis occurs mainly in cells as reduced mitochondrial volume, increased bilayer membrane density and reduction or disappearance of mitochondrial cristae [6]. Biochemically, there is intracellular glutathione (GSH) depletion and decreased glutathione peroxidase 4 (GPX4) activity, resulting in a large amount of ROS, which promotes ferroptosis [7]. Generally, ferroptosis is a biological process regulated by multiple genes.

Although the contribution of ferroptosis to physiology remains obscure, its role in a variety of human pathological states, such as neurotoxicity, traumatic brain injury, and malignancies, has been confirmed [8, 9]. Previous studies have analyzed the role of ferroptosis in malignancies from different perspectives. On the one hand, some studies believe that ferroptosis might suppress the immune system and allow part of tumor cell growth because it affects antitumor immunity by cooperating with multiple different cell types present in the tumor microenvironment [10]. On the other hand, research has also suggested that ferroptosis can be induced by tumor microenvironment-responsive multistaged liposomes through amplifying oxidative stress to inhibit tumor growth and cancer cell proliferation [11].

Ferroptosis has gradually shown attractive antitumor effects. The activation of malignant tumor cell death or growth restriction caused by different ferroptosis inducers or induction methods confirms its inhibitory influence on malignancies. For example, inhibition of sigma 1 receptor (S1R), which is heavily expressed in hepatocytes, can promote ferroptosis in hepatocellular carcinoma (HCC) cells [12]. In triple-negative breast cancer (TNBC) cells, inhibition of activation of the MUC1-C/xCT signalling pathway can induce ferroptosis [7].

In previous cases, a constructed model based on ferroptosis-associated genes can be used for prognostic prediction in malignancies and provide ideas for us to take a closer look [13–16]. This study attempted to establish and verify a prognostic prediction model based on ferroptosis-associated DEGs from the TCGA database. Additionally, we investigated the correlation between the tumor immune microenvironment, including immune cell infiltration and the enrichment of immune-related pathways, with the two risk groups that were stratified based on the risk score of this model.

## 2. Materials and methods

### 2.1. Data preparation

The RNA sequencing (RNA-seq) dataset and related clinical data of 502 LUSC samples, including sex, age, grade, status, and stage, were downloaded from the TCGA database (December 21, 2020) (https://portal.gdc.cancer.gov/repository) as the training cohort. The RNA-seq dataset and clinical data of another 249 tumor patients from the GEO database (GSE157009) were downloaded for the testing cohort (https://www.ncbi.nlm.nih.gov/gds) using the keywords “lung cancer”, “LUSC”, and “lung squamous cell carcinoma”, “Homo sapiens”, and “Expression profiling by array”. In addition, the information from TCGA and GEO are both open source. Therefore, this study was exempted from permission from ethics committees. To analyze the deidentified data from TCGA and GEO, institutional review and approval were accessed based on the policies and publication guidelines. The "limma" R package with a Voom method was employed to normalize the RNA-seq data of each LUSC sample. Sixty ferroptosis-associated genes were taken from previous studies [17–20], and all of them are listed in S1 Table.

### 2.2. Construction and validation of a prognostic ferroptosis-associated gene signature

The "limma" package of R software was applied to distinguish the differentially expressed genes (DEGs) between two groups (tumor tissues versus adjacent nontumorous tissues) with a false discovery rate (FDR) less than 0.05 in the TCGA database. Herein, univariate Cox regression analysis was employed to examine the relationship between ferroptosis-associated genes and overall survival (OS) of samples in the TCGA database. The Benjamini & Hochberg (BH) correction method was used to further calibrate the P values. To achieve the minimum risk of overfitting, we adopted LASSO [21, 22] to build a prognostic model. LASSO was applied for the selection and shrinkage of variables by using the packages "glmnet" and "survival" from R software. The independent variable in the regression was the normalized expression matrix of candidate prognostic DEGs. The response variables correspond to the OS and status of samples in the TCGA database. The penalty parameter (λ) for the signature can be obtained by tenfold cross-validation based on the minimum criteria. For example, the λ value corresponding to the lowest partial likelihood deviance has the same calculating method. The calculation of risk scores acquires two kinds of parameters. One is the normalized expression level of each gene, and the other includes the related regression coefficients. The formula of the risk score can be given as follows: risk score = *e*^sum(each gene’s expression × corresponding coefficient)^. The samples can be classified into two groups (high risk and low risk) according to the median value of the risk score. Based on the expression of genes in this model, the most commonly used dimensionality reduction method PCA was employed with the "prcomp" and "ggplot2" functions of the "stats" R package. Different from PCA, a nonlinear dimensionality reduction method t-SNE was used to investigate the distribution of the two groups by using the "Rtsne" package. For the survival analysis, the optimal cutoff value of every gene expression related to ferroptosis is crucial for sample grouping, which can be obtained using the "survival" and "survminer" R packages. Finally, the "survivalROC" package was applied to perform ROC curve analysis to evaluate the effectiveness of the prognostic prediction model.

### 2.3. Protein–protein interaction (PPI) network analysis

An interaction network showing the association of DEGs related to prognosis was formed from the STRING database. The STRING database (https://string-db.org/) includes a function for predicting protein interactions and reveals interactions with genes. Each PPI in the interaction network has ’scores’ that determine the reliability of association. Thus, the interaction association can be obtained by PPI network analysis. The score varies from 0 to 1, where 1 means the highest reliability; in contrast, 0 means no correlation. Central genes were distinguished by using Cytoscape with a threshold of the highest reliability of 0.50.

### 2.4. Functional enrichment analysis

GO, as a primary bioinformatics method for annotation of genes and analysis of their biological processes, consists of the following separate parts: molecular function (MF), biological process (BP), and cellular component (CC). The KEGG (Kyoto Encyclopedia of Genes and Genomes) database is used to illustrate advanced functions and biological systems from large-scale molecular datasets generated by high-throughput experimental technologies, which systematically analyze gene function and link genomic information and functional information, including pathway databases, hierarchical classification databases, gene databases, genome databases, etc., and its pathway database is the most widely used public database of metabolic pathways. Four packages, ’clusterProfiler’, ’enrichplot’, ’org.Hs.eg.db’ and ’ggplot2’, were employed to conduct GO and KEGG enrichment analyses based on the DEGs (|log2FC| ≥ 1, FDR < 0.05) between the high- and low-risk groups. Finally, p values were calibrated using the BH method through R software.

### 2.5. Gene set enrichment analysis (GSEA)

In this study, the reference gene set “c2.cp.kegg.v6.2.symbols.gmt” can be acquired from the supplementary files of previous papers or downloaded from the online open source Molecular Signatures Database (MSigDB) (http://software.broadinstitute.org/gsea/msigdb). We identified significant pathways enriched in each of the 5 ferroptosis-associated genes by GSEA. Gene set operations were repeated 1,500 times for each analysis, and the expression levels of 5 ferroptosis-associated genes were set as phenotypes. The nominal p value and normalized enrichment score (NES) were both applied to the pathway enrichment analysis. GSEA has the following crucial statistics: the NES, enrichment score (ES), FDR and p value. A pathway was considered significantly enriched when the p value was less than 0.05 while the FDR was less than 0.25.

### 2.6. Single-sample gene set enrichment analysis (ssGSEA)

We used ssGSEA to evaluate the infiltration score of 16 immune cells and the activity of 13 immune-related pathways through the "limma", "gsva", "GSEABase", "XML", "graph", and "annotate" packages [23]. A log 2-fold change in gene expression profiles was calculated between the high- and low-risk groups, and the different immune statuses between the two groups were compared. Differences with an FDR-adjusted P < 0.05 were regarded as effective. We displayed the annotated gene set file in S2 Table.

### 2.7. Statistical analysis

Student’s t test was adopted to compare the gene expression between tumor tissues and normal tissues. ANOVA + post-hoc was employed for the relationship between the genes and different clinical characteristics. The Wilcoxon signed-rank test was used to determine the expression of the 5 ferroptosis-associated genes in LUSC tissues and matched adjacent normal tissues. Differences in proportions were compared by the chi-squared test. LASSO Cox proportional hazards regression analysis was conducted by penalized package. The Mann–Whitney test with P values adjusted by the BH method was used to compare the ssGSEA scores of immune cells or pathways between the high- and low-risk groups. Kaplan–Meier analysis was performed using the log-rank test to compare differences between the two groups. Univariate and multivariate Cox regression analyses were implemented to identify independent predictors of OS. All statistical analyses were performed with R software (Version 3.6.3) or R software (Version 4.0.3). If not specified above, a P value less than 0.05 was considered statistically significant, and all P values were two-tailed.

## 3. Results

### 3.1. Identification of prognostic ferroptosis-associated DEGs in the TCGA cohort

**Fig 1:**
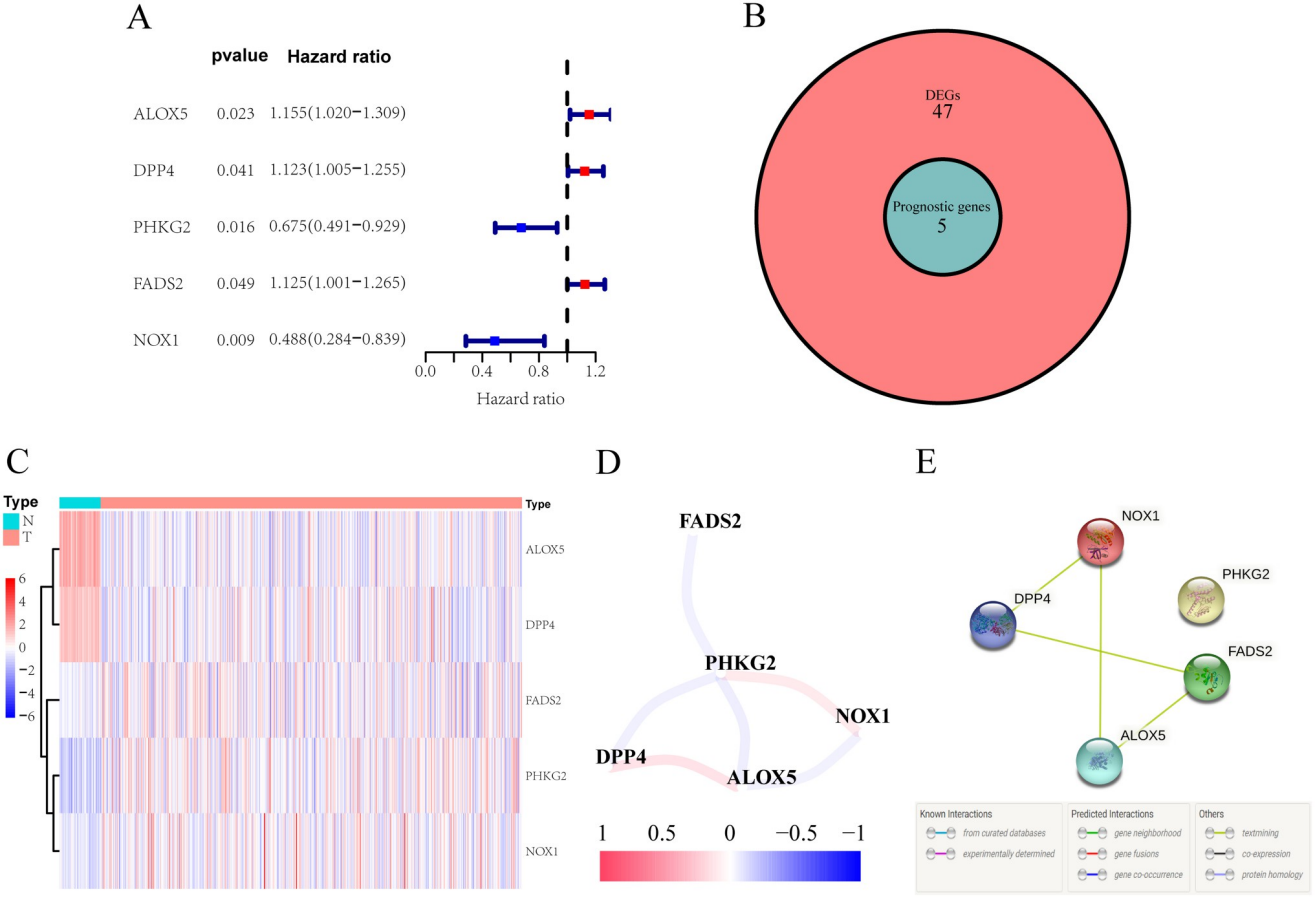
Identification of the candidate ferroptosis-associated genes in the TCGA cohort. A. Forest plots showing the results of the univariate Cox regression analysis between gene expression and OS. B. Venn diagram to identify differentially expressed genes between tumor and adjacent normal tissue intersect with OS-correlated genes. C. The 5 overlapping genes were all difference expression between normal and tumor tissue. D. The correlation network of candidate genes. The correlation coefficients are represented by different colors (positive correlation is red, negative correlation is blue). E. The PPI network downloaded from the STRING database indicated the interactions among the candidate genes.

**Fig 2:**
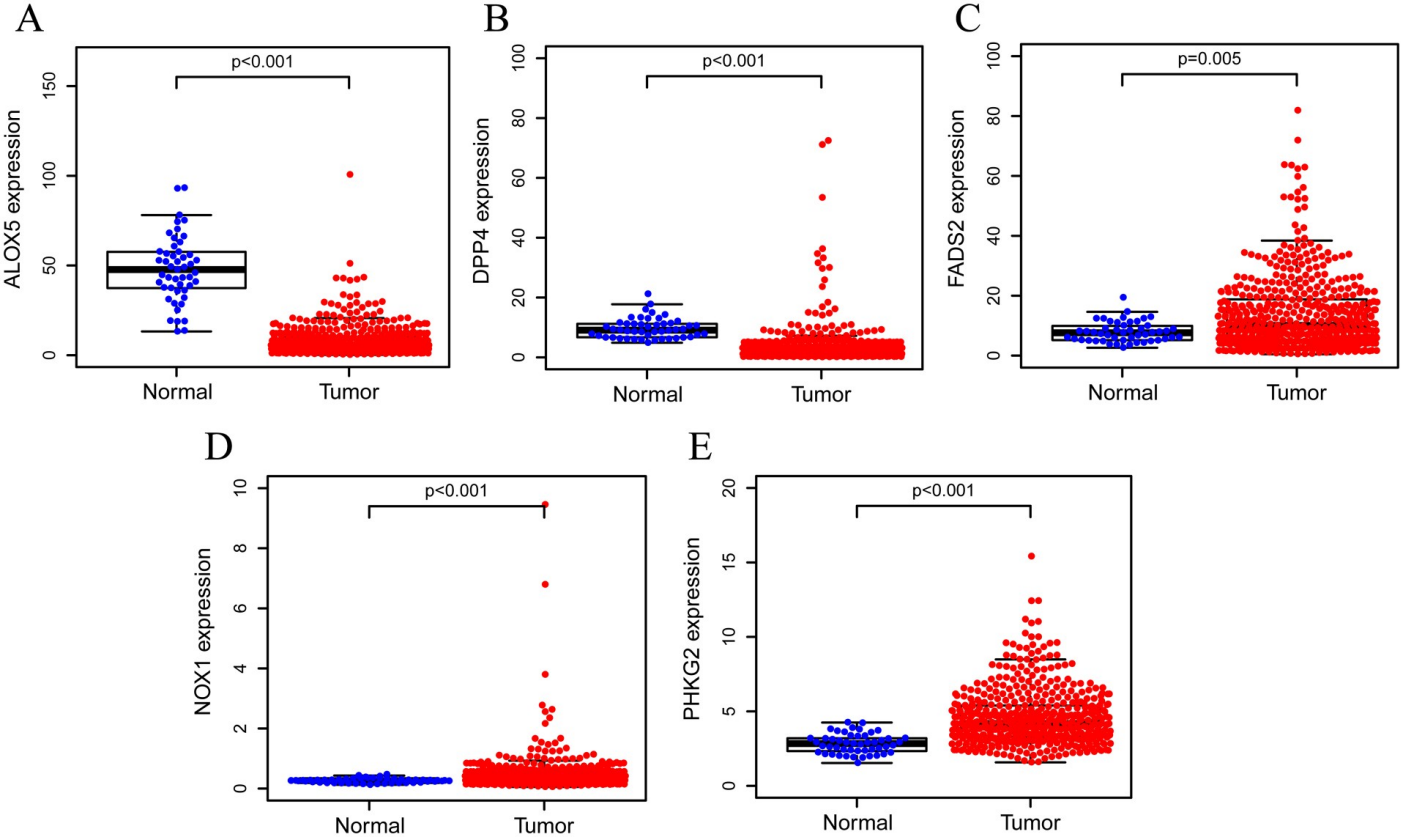
The expression of ALOX5(A) and DPP4(B) are significantly lower in LUSC tissues than in normal tissues and the expression of FADS2(C), NOX1(D), and PHKG2(E) are opposite in the TCGA cohort. (all adjusted P<0.05).

**Fig 3:**
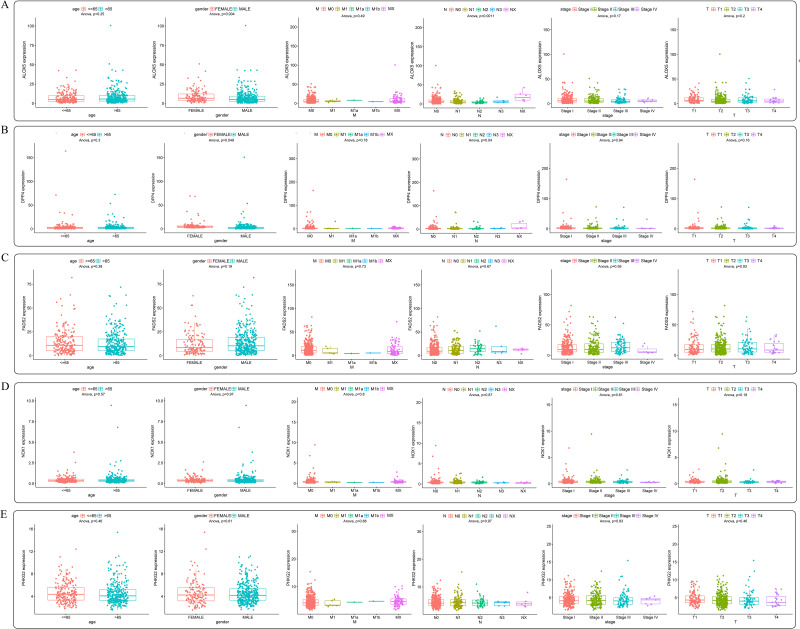
The correlation of 5 ferroptosis-associated genes expression with clinicopathological features in the TCGA cohort.

A total of 502 LUSC patients’ genes expression and clinical information from the TCGA cohort and 247 LUSC patients’ genes expression from the GEO cohort were enrolled. The detailed clinical characteristics of these patients are summarized in Table 1. We obtained 60 ferroptosis-associated genes through previous research; 52 DEGs were differentially expressed between tumor and normal tissues, and 5 of them were correlated with OS in the univariate Cox regression analysis in the TCGA cohort (Fig 1A). Taking the intersection of the 52 DEGs and 5 prognosis-related genes from the previous step, a total of 5 prognosis- and ferroptosis-associated DEGs were screened out (Fig 1B). The heatmap shows the differential expression of the 5 genes between tumor and normal tissues. ALOX5 and DPP4 were significantly higher in normal tissues, while FADS2, NOX1, and PHKG2 were significantly higher in LUSC tissues (Fig 1C). The interaction network among these 5 genes shows that there is a certain correlation between them, and the correlation coefficient is 0.4 (Fig 1D). PPI network analysis was performed using the online database STRING with an interaction score of 0.4 as the threshold. There were 4 connections and 4 nodes, further presenting the correlation between the 5 genes (Fig 1E). We used the Wilcoxon-rank sum test to analyze the relationship between the expression of the 5 ferroptosis-associated genes in different tissues, and the results showed that the expression of the 5 ferroptosis-associated genes was significantly different in LUSC tissues and normal tissues in the TCGA cohort. The results showed that ALOX5 and DPP4 were significantly higher in normal tissues, while FADS2, NOX1, and PHKG2 were significantly higher in LUSC tissues (all adjusted P<0.05) (Fig 2). Subsequently, we used the Wilcoxon signed-rank test to determine the expression of 5 ferroptosis-associated genes in 46 LUSC tissues and matched adjacent normal tissues in the TCGA cohort. The expression of ALOX5 and DPP4 was significantly higher in 46 matched adjacent normal tissues than in LUSC tissues. In contrast, FADS2, NOX1, and PHKG2 were significantly higher in matched adjacent normal tissues, which is consistent with the previous unpaired analysis results (all adjusted P<0.05) (S1 Fig). Because these 5 genes were significantly related to OS performed by univariate Cox regression analysis (Fig 1A), Kaplan–Meier survival analysis was performed to examine the influence of different expression levels of these 5 genes on the prognosis of LUSC. The results showed that patients with low PHKG2 expression experienced a shorter OS duration than those with high PHKG2 expression (P = 0.026), and the patients with high DPP4 expression experienced a shorter OS duration than those with low DPP4 expression (P = 0.043) (S2 Fig). We also explored the relationship between the 5 genes and different clinical characteristics. Among those 5 genes, the expression of ALOX5 was obviously correlated with part of the T stage and N stage and sex; the expression of DPP4 was obviously correlated with part of the N stage, and the expression of NOX1 was obviously correlated with part of the T stage (all adjusted P<0.05) (Fig 3).

**Table 1:** Clinical characteristics of the LUSC patients used in this study.

	TCGA cohort	Merge cohort
No. of patients	493	740
Age (median, range)	68(39–85)	66 (37–75)
**Gender (%)**		
Female	128(26%)	180 (24%)
Male	365(74%)	560 (76%)
**Stage (%)**		
I	241	351
II	158	237
III	83	124
IV	7	17
unknown	4	11
**Survival status**		
OS days(median)	660	620

### 3.2. Construction of a prognostic model in the TCGA cohort

**Fig 4:**
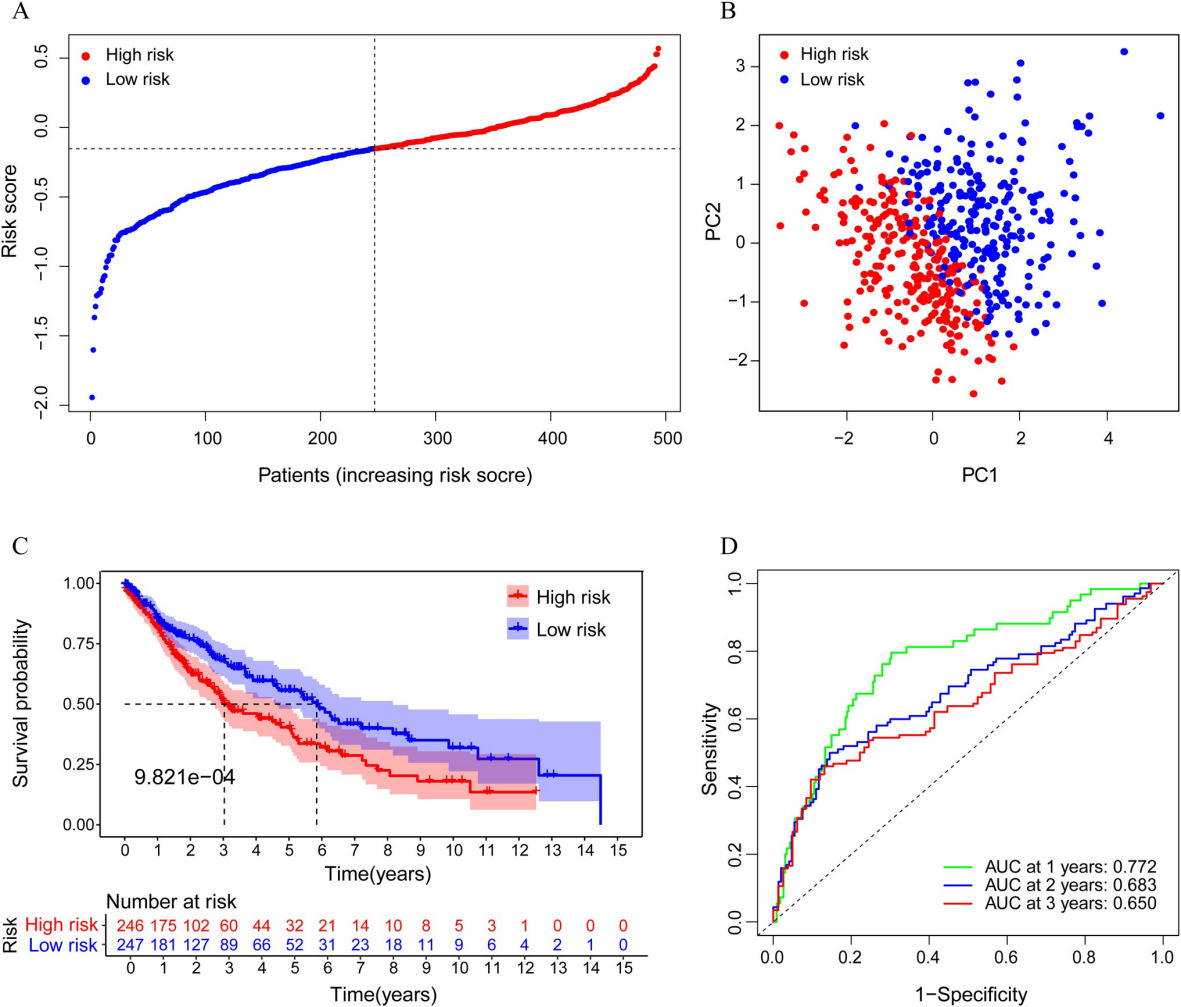
Prognostic analysis of the 5 gene signature model in the TCGA cohort. A. The distribution by median value of the risk scores with this model. B. PCA plot. C. Kaplan-Meier curves for the OS of patients in the high-risk group and low-risk group. D. AUC of time-dependent ROC curves verified the prognostic performance of the risk score.

We further used LASSO Cox regression analysis to minimize the risk of overfitting. A risk model based on these 5 ferroptosis-associated gene expression profiles was selected in LASSO; thus, the predictive accuracy could be significantly improved. In detail, a 5-gene signature was identified based on the optimal value of λ (S3 Fig). The optimal cutoff expression value of each gene indicated that high expression of ALOX5, DPP4, and FADS2 is correlated with a poor prognosis, while NOX1 and PHKG2 are the opposite. The risk score was calculated as follows: e [0.116 * expression level of ALOX5 + 0.037 * expression level of DPP4 + (-0.306) * expression level of PHKG2 + 0.122 * expression level of FADS2 + (-0.526) * expression level of NOX1]. The patients were stratified into a high-risk group (n = 246) or a low-risk group (n = 247) according to the median value of the risk score (Fig 4A). The high-risk group was found to be significantly associated with higher TNM stage and higher age in the TCGA cohort (Table 2). To verify whether the model can effectively distinguish patients in different groups, we realized visualization through dimensionality reduction processing. PCA analysis indicated that the patients in different risk groups were distributed in two directions (Fig 4B). Consistently, the Kaplan–Meier curve indicated that patients in the high-risk group had a significantly worse OS than their low-risk counterparts (Fig 4C, P< 0.001). The predictive performance of the risk score for OS was evaluated by time-dependent ROC curves. The area under the curve (AUC) reached 0.772 at 1 year, 0.683 at 2 years, and 0.650 at 3 years, proving the performance of this prognostic model (Fig 4D).

**Table 2:** Baseline characteristics of the patients in different risk groups.

Characteristics	TCGA cohort		
	High risk	Low risk	P value
Gender (%)			0.169
Female	65 (26.4)	63 (25.5)	
Male	181 (73.6)	184 (74.5)	
Age (%)			0.042
< 60y	48 (19.5)	42 (17.0)	
≥ 60y	196 (79.7)	202 (81.8)	
unknown	2 (0.8)	3 (1.2)	
TNM stage (%)			0.012
I+II	199 (80.9)	200 (81.0)	
III+IV	44 (17.9)	46 (18.6)	
unknown	3 (1.2)	1 (0.4)	

### 3.3. Validation of the 5-gene signature in the GEO and TCGA cohorts

**Fig 5:**
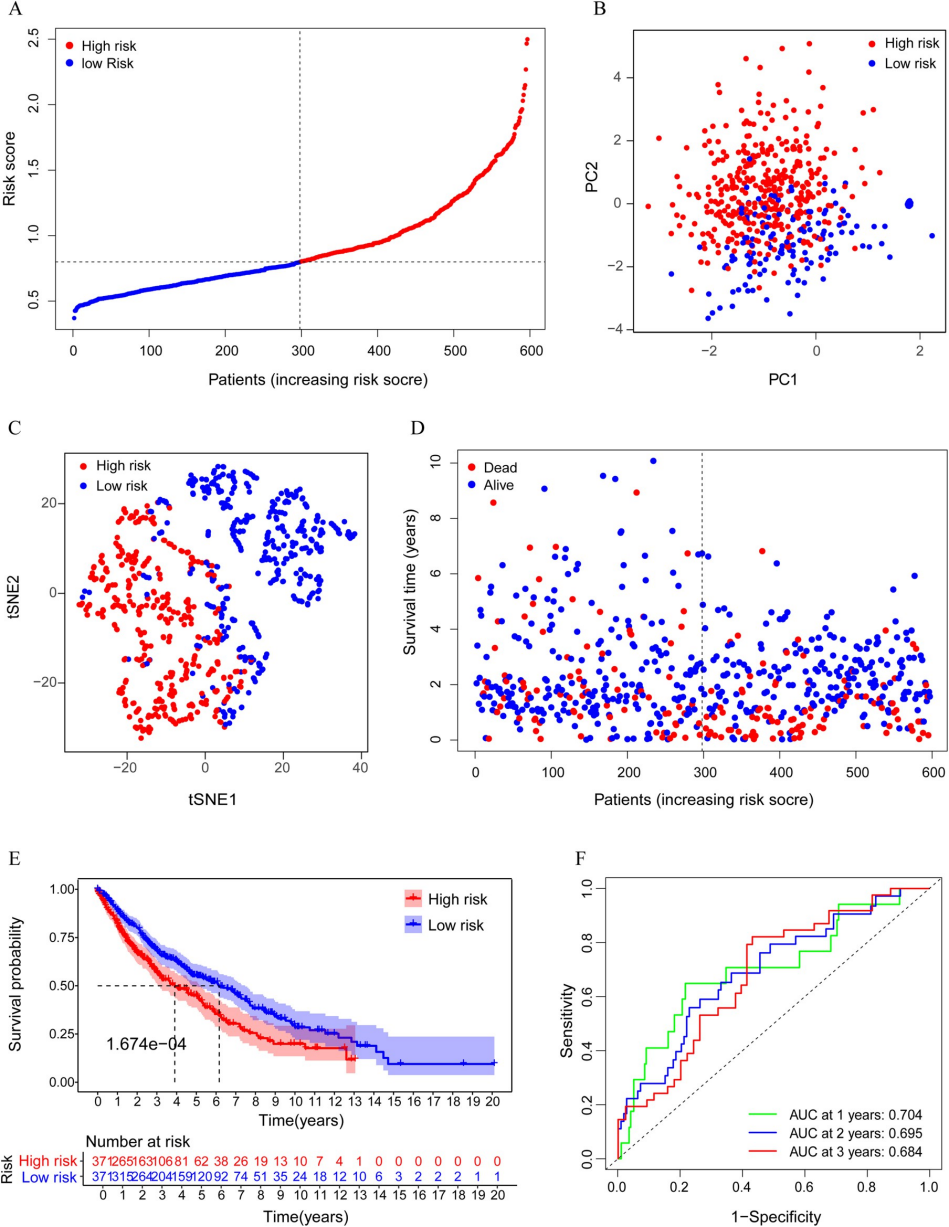
Prognostic analysis of the 5-gene signature model in the GEO cohort. A. The distribution by median value of the risk scores with this model. B. PCA plot. C. t-SNE analysis. D. The distributions of OS status, OS and risk score. E. Kaplan-Meier curves for the OS of patients in the high-risk group and low-risk group. F. AUC of time-dependent ROC curves verified the prognostic performance of the risk score.

We tested the robustness of the model constructed by a cohort merged from the GEO dataset and TCGA database. The patients from the merged cohort were also categorized into two risk groups by the median value calculated with the same formula as that from the TCGA cohort (Fig 5A). Similar to the results obtained from the TCGA cohort, PCA and t-SNE analysis confirmed that patients in the two subgroups were distributed in discrete directions (Fig 5B and 5C). Likewise, patients in the high-risk group encountered death earlier (Fig 5D) and had a reduced survival time compared with those in the low-risk group (Fig 5E, P<0.05). In addition, the AUC of the model was 0.704 at 1 year, 0.695 at 2 years, and 0.684 at 3 years (Fig 5F), proving that this prediction model can effectively predict the prognosis of patients further.

### 3.4. Independent prognostic value of the 5-gene signature

**Fig 6:**
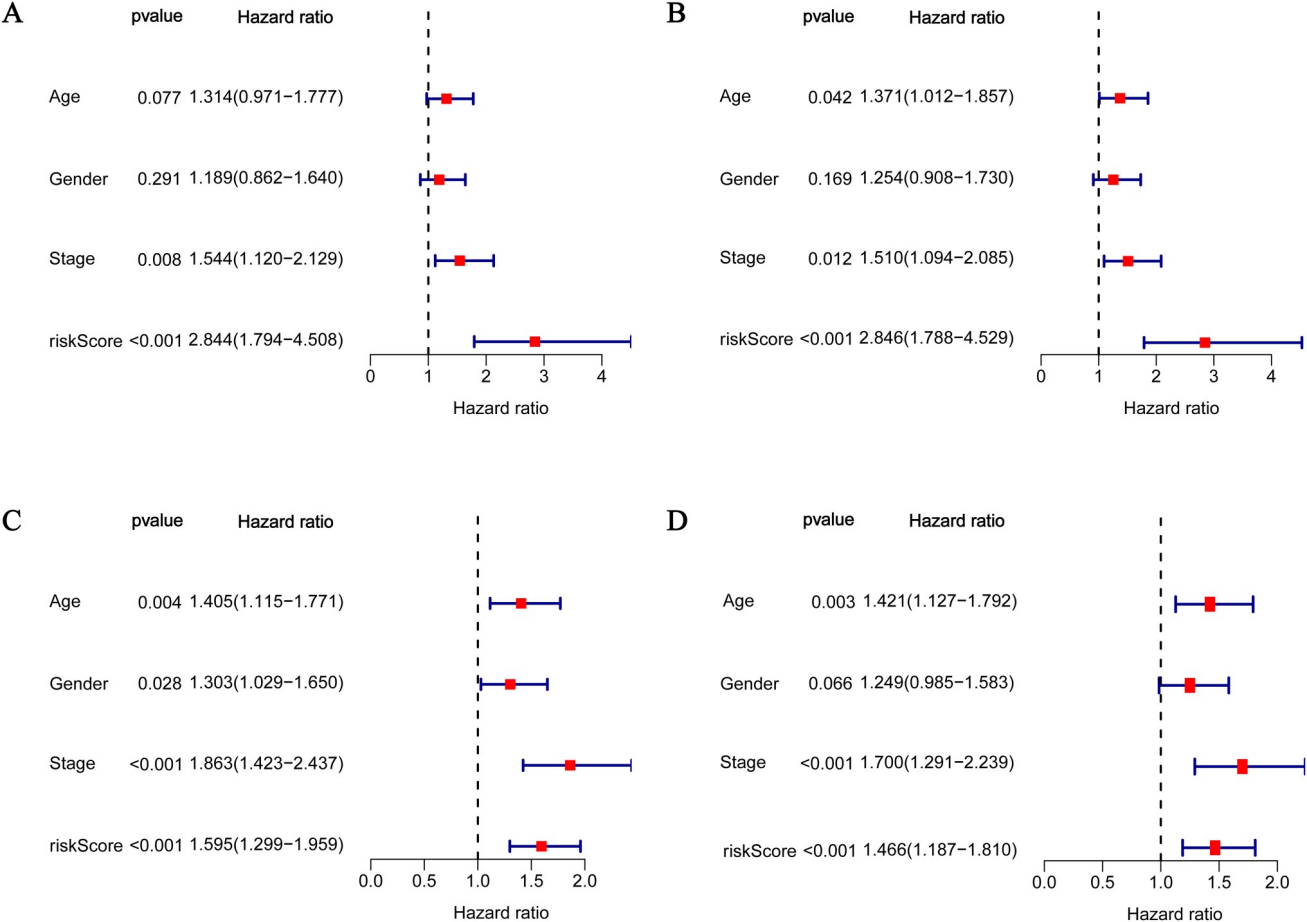
Results of the univariate and multivariate Cox regression analyses regarding OS in the TCGA cohort (A, B) and the GEO cohort (C, D).

Univariate and multivariate Cox regression analyses were carried out among the available variables to determine whether the risk score was an independent prognostic predictor for OS. In univariate Cox regression analyses, the risk score was significantly associated with OS in the TCGA and merged cohorts (HR = 2.844, 95% CI = 1.794–4.508, P< 0.001; HR = 1.595, 95% CI = 1.299–1.959, P< 0.001) (Fig 6A and 6C). After correction for other confounding factors, the risk score still proved to be an independent predictor for OS in the multivariate Cox regression analysis (HR = 2.846, 95% CI = 1.788–4.529, P< 0.001; HR = 1.466, 95% CI = 1.187–1.810, P< 0.001) (Fig 6B and 6D).

### 3.5. GO enrichment and KEGG pathway analyses

**Fig 7:**
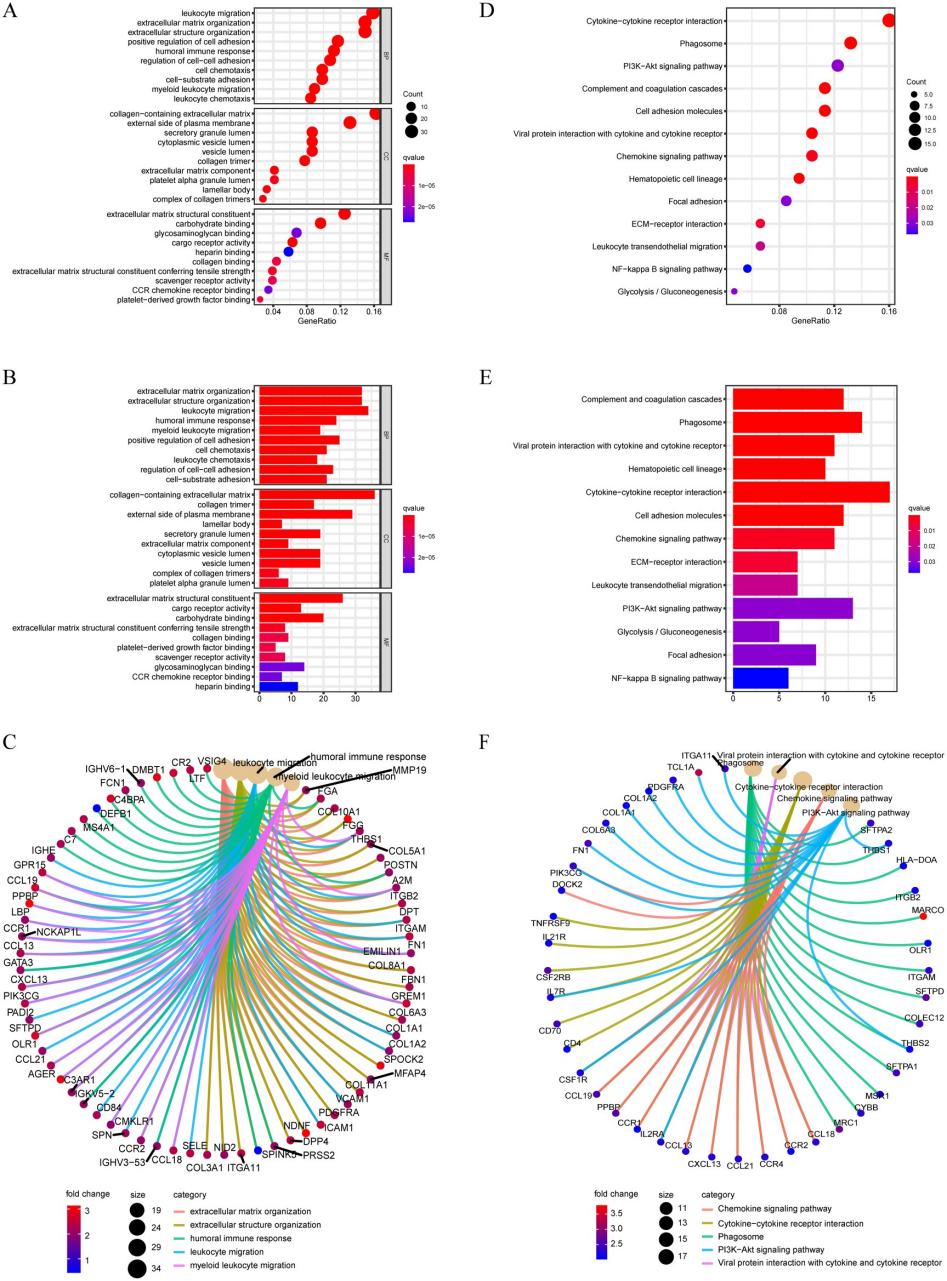
Results of GO (A, B) and KEGG analyses (D, E). The circle plots show the enrichment relationship between genes and the main enriched terms in GO (C) and KEGG analyses (F) in the TCGA cohort.

**Fig 8:**
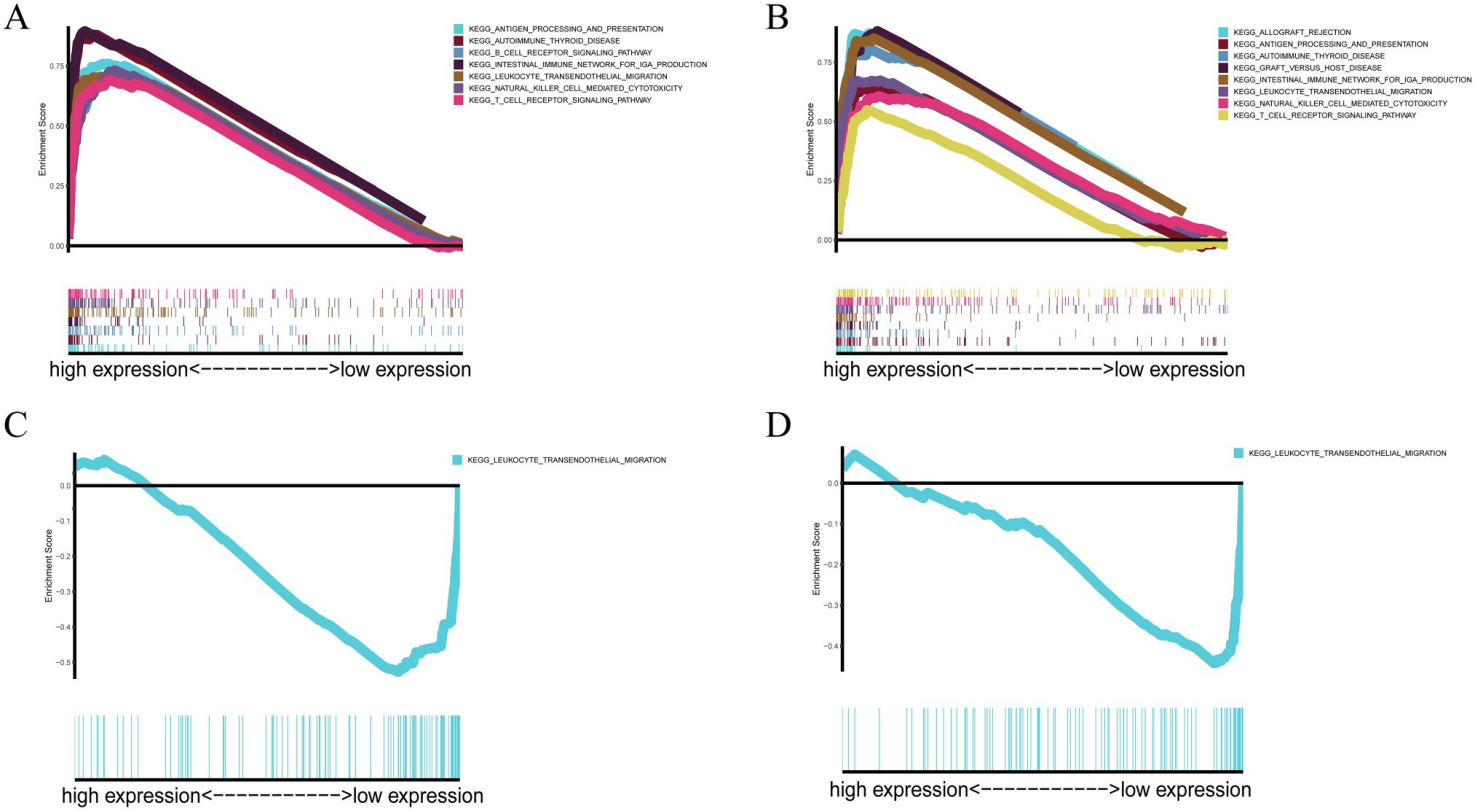
Multiple GSEA enrichment plots of ALOX5(A); DPP4(B); NOX1(C); PHKG2(D) in the TCGA cohort. NES, normalized enrichment score; ES, enrichment score; FDR, false discovery rate.

**Fig 9:**
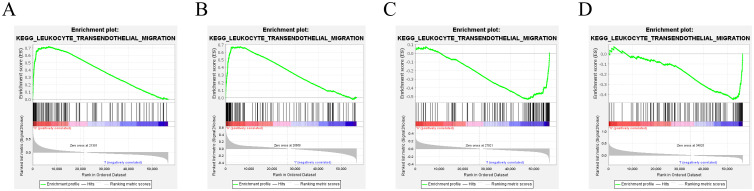
Gene set enrichment analysis confirmed that 4 genes in this model including ALOX5(A), DPP4(B), NOX1(C), and PHKG2(D) are all enriched in leukocyte transendothelial migration.

To elucidate the biological functions and pathways that were associated with the risk score, the DEGs were used to perform GO enrichment and KEGG pathway analyses in TCGA cohort. The results showed that various pathways, including phagosomes, pyrimidine metabolism, viral protein interactions with cytokines and cytokine receptors, and cytokine–cytokine receptor interactions, especially some immune-related pathways, such as the leukocyte transendothelial migration pathway, were significantly enriched in the cohort by KEGG pathway analyses (all adjusted P<0.05, Fig 7D–7F). Interestingly, the DEGs were also obviously enriched in many immune-related biological processes, including leukocyte migration, humoral immune response, myeloid leukocyte migration, and leukocyte chemotaxis, through GO enrichment (all adjusted P<0.05, Fig 7A–7C). Taking into account the correlation between ferroptosis and immunity, enrichment analysis provides evidence that the model may also be related to immunity. Thus, to further confirm the above speculation, GSEA was performed to reveal differences between the high and low expression cohorts of 5 ferroptosis-associated genes respectively. The results showed that multiple immune-related pathways were significantly enriched (Fig 8), and the most significantly enriched signalling pathway was the leukocyte transendothelial migration pathway in the high ALOX5 and DPP4 and low NOX1 and PHKG2 expression phenotypes, which was consistent with the results of the previous KEGG pathway analyses (Fig 9).

### 3.6. Single-gene immune analysis

**Fig 10:**
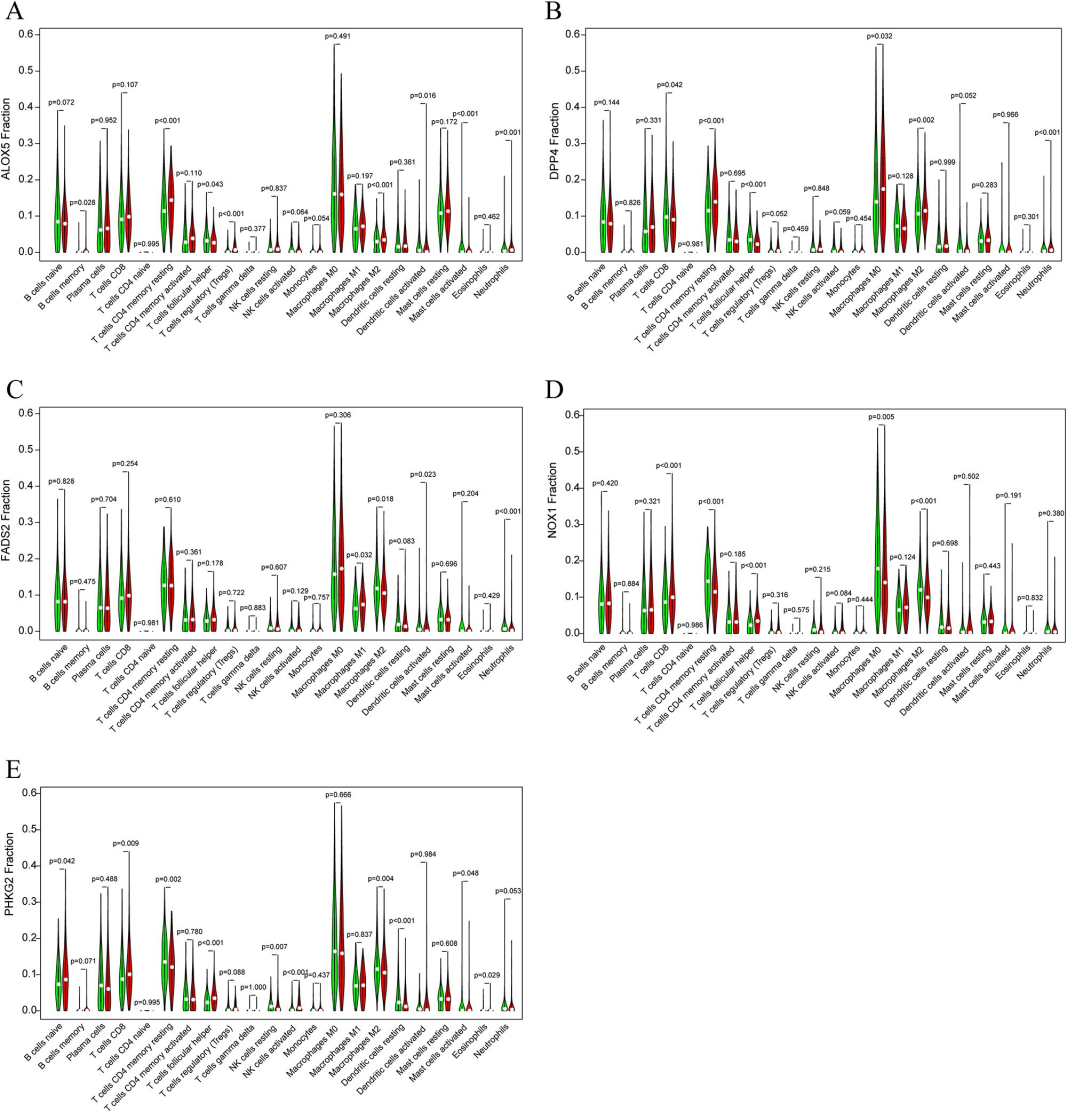
Violin plot showed the proportions of 22 immune cells between high risk and low risk with the 5 genes.

Because of the significant association with immunity in our model, we extracted the expression levels of these 5 genes in tumor samples, divided the samples into high- and low-risk groups based on the median value, and then compared the differences in the content of each immune cell in the two groups by CIBERSORT. The most significant immune cells related to each of the 5 ferroptosis-associated genes were filtered out (Fig 10A–10E).

### 3.7. Immune cell infiltration in different risk groups

**Fig 11:**
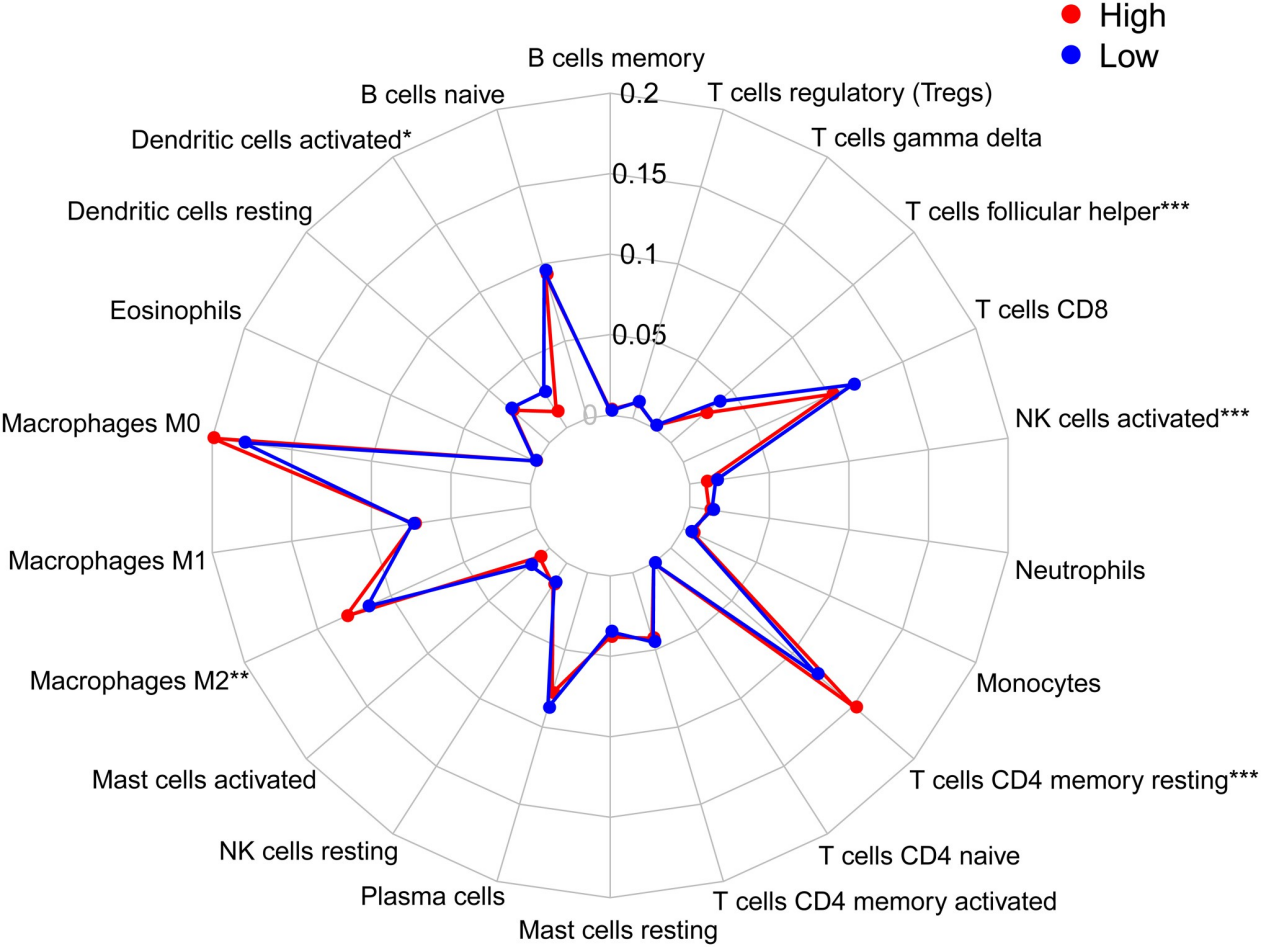
The radar charts depict a comparative summary of various immune cells in these two risk groups (*P< 0.05, **P< 0.01, ***P< 0.001).

**Fig 12:**
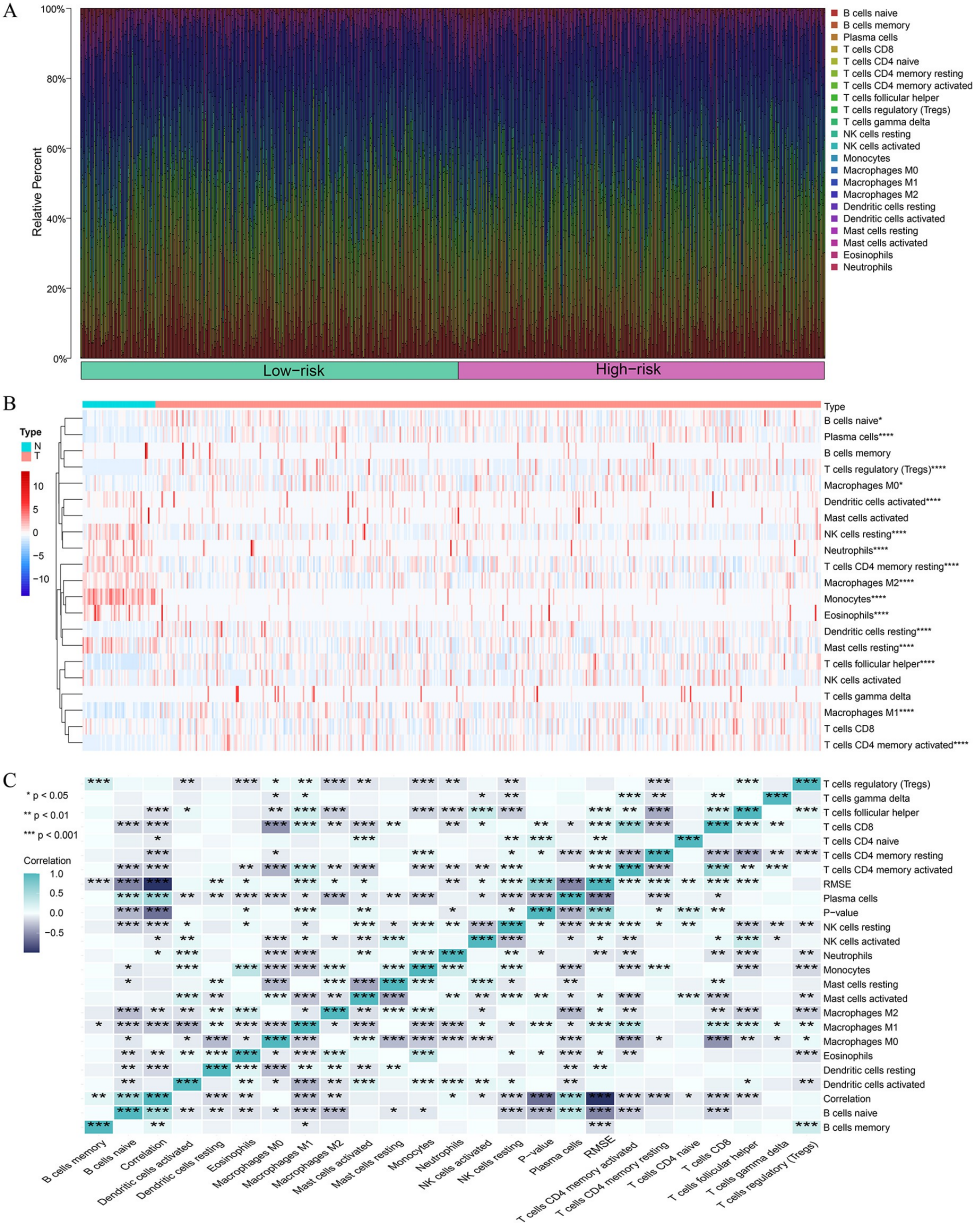
The relationship between the genes and immune cell infiltration in the TCGA cohort. A Bar plot showed the proportion of 22 immune cells with each sample. B. The heat map showed the level of immune cell infiltration of each sample between normal tissues and LUSC tissues. C. Correlation matrix of all 22 immune cells proportions.

**Fig 13:**
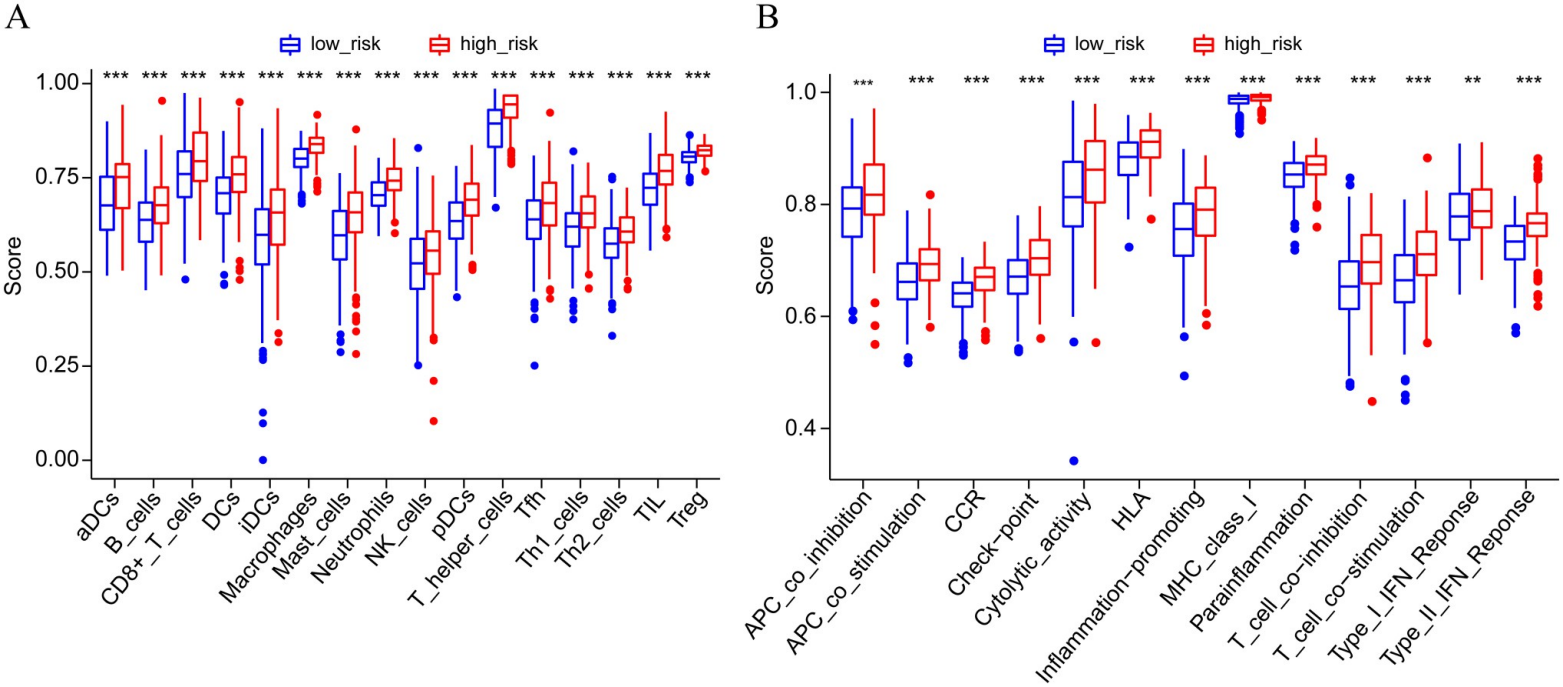
Comparison of the ssGSEA scores between different risk groups in the TCGA cohort. The scores of 16 immune cells (A) and 13 immune-related functions (B) are displayed in boxplots. CCR, cytokine-cytokine receptor. Adjusted P values were showed as: ns, not significant; *, (P< 0.05; **, P< 0.01; ***, P< 0.001).

Previous studies have revealed that tumor-infiltrating immune cells are related to prognosis [24]. To find more evidence to support the correlation between the high- and low-risk groups and immune infiltration, we explored the infiltration of specific tumor immune cell subsets by CIBERSORT to obtain the abundance of the 22 immune cell types in different groups. The radar charts depict a comparative summary of various immune cells in the two risk groups (Fig 11). We found that M2 macrophages and resting memory CD4 T cells were significantly highly expressed in the high-risk group, while follicular helper T cells, activated NK cells, and activated dendritic cells were significantly higher in the low-risk group (S4 Fig). Therefore, we further analyzed differences in immune infiltration between the tumor and normal tissues for 22 immune cell types using CIBERSORT. First, we presented the proportion of each immune cell in all samples using a bar plot (Fig 12A) and then used a heatmap to compare the levels of immune cell infiltration between normal and LUSC tissues (Fig 12B). Correlation was observed in various immunocyte subpopulations (Fig 12C). To further explore the correlation between the risk score and immune status, we quantified the enrichment scores of diverse immune cell subpopulations, related functions or pathways with ssGSEA. To our surprise, all of them, including immune cell subpopulations such as dendritic cells, B cells, CD8+ T cells, macrophages, mast cells, neutrophils, NK cells, helper T cells, tumor-infiltrating lymphocytes, and Tregs, and related functions or pathways such as antigen-presenting cell (APC) coinhibition, APC costimulation, chemokine receptor, checkpoint, cytolytic activity, human leukocyte antigen, inflammation-promoting, major histocompatibility complex (MHC) class I, parainflammation, T-cell coinhibition, T-cell costimulation, and type I and type II interferon (IFN) response, had higher scores in the high-risk groups (Fig 13, adjusted P< 0.001). This result reminds us that in LUSC, the ferroptosis-associated gene signature we constructed is significantly correlated with immune infiltration. The results shown above all remind us that there are significant differences in the immune status of the high- and low-risk groups with this model in LUSC.

## 4. Discussion

LUSC, a malignancy associated with high mortality, is the leading cause of cancer death in 93 countries, and most countries are still observing a rising incidence of lung cancer [1]. To this end, there is an urgent need to develop a prognosis prediction model as a useful tool for prognosis prediction and then provide guidance for clinical practice, perhaps reveal important information and conduct more in-depth mechanism discussions, and then develop some new and effective treatment methods or targets.

In this study, we obtained 5 ferroptosis- and prognosis-associated DEGs from 52 ferroptosis-associated DEGs and 5 prognosis-related genes by univariate Cox analysis in LUSC from the TCGA database and then established a prognostic model using the expression profile of the 5 genes through LASSO Cox regression analysis and validated it in an external cohort to ensure authenticity and validity. The predictive power of the model was evaluated by ROC curve analyses. The successful establishment of this model suggests that there is a certain correlation between ferroptosis and the prognosis of LUSC patients. Ferroptosis is a common event in almost all types of malignancies. We also explored the relationship between ferroptosis and other cancers. Breast cancer and hepatocellular carcinoma data from the TCGA database were obtained to repeat the work of this study and find that ferroptosis-related genes also have a significant relationship with prognosis in breast cancer and hepatocellular carcinoma. This finding strengthens the view that ferroptosis-related genes can be regarded as a prognostic signature for cancers (S5 and S6 Figs). When we reviewed the 5 genes in the model further, we found that these 5 genes can be divided into two categories, including oxidant metabolism (ALOX5, NOX1, PHKG2) and lipid metabolism (DPP4, FADS2). These two types of genes play an important role in the occurrence and development of malignant tumors, which also confirms the scientific nature of our model in a certain way.

Ferroptosis, as a new form of programmed cell death, is mainly characterized by the accumulation of reactive oxygen species (ROS) resulting from iron accumulation and lipid peroxidation [5]. Past research has shown that tumor cells can be induced to ferroptosis in many ways and then inhibit tumor growth. Furthermore, ferroptosis can trigger inflammation-associated immunosuppression in the tumor microenvironment, thus favoring tumor growth. In addition, previous studies have also shown that ferroptosis is also closely related to immunity and plays different roles in tumor cells through immunomodulation. First, ferroptosis exerts a cancer-promoting role in the immune system by suppressing antitumor immunity or causing its deficiency; for example, ferroptotic cells can release some signals that affect antigen-presenting cells (APCs) and other immune cells [25, 26]. Ferroptosis more often plays an anticancer role in the immune system, and studies have revealed that ferroptosis can inhibit cancer cell proliferation and tumor growth by regulating the tumor microenvironment (TME) and mediating tumor suppressive activity [27].

Although studies have shown that ferroptosis has a certain significance with immunity in tumorigenesis and development, it is still unclear whether it plays the same important role in LUSC. The occurrence and development of LUSC are significantly related to immunity [28]. In our study, DEGs were enriched in several immune-related biological processes, such as leukocyte migration, myeloid leukocyte migration and leukocyte chemotaxis, according to GO enrichment. Interestingly, we also found that these biological processes all act on the leukocyte transendothelial migration pathway [29], which was indicated by KEGG pathway analyses and was consistent with the results of our previous single-gene enrichment analysis. Trafficking of leukocytes is a key process for immune cell development and host defense. Leukocyte transendothelial migration (TEM) is a vital physiological process that occurs during both the adaptive and innate immune response, and routine immune surveillance can further cause the occurrence of cancer [30]. As the largest cells of the leukocyte family, particularly macrophages, their infiltration often correlates with the aggravation of several diseases, including cancers. The migration of macrophages across tissues can ensure efficient tissue infiltration [31]. Immune correlation analysis showed that M2 macrophages were significantly highly expressed in the high-risk group of this model; coincidentally, single-gene immune cell infiltration analysis showed that almost all 5 genes were significantly related to immune cells, especially M2 macrophages. M2 macrophages, immune suppressor cells, can release suppressive factors such as ROS to suppress T and NK-cell functions and promote tumor growth and metastasis [32, 33]. Based on the above analysis, we speculate that M2 macrophages are regulated by the leukocyte transendothelial migration pathway to ensure their effective infiltration, thereby playing an immunosuppressive role in the immune microenvironment and ultimately promoting tumor development in LUSC.

Moreover, many related functions or pathways, such as type I and type II IFN responses and parainflammation, also have higher scores in the high-risk group; they can play different roles in tumorigenesis and development, such as promoting tumor growth and proliferation and migration to metastatic sites [34], evading immunosurveillance [35] and inducing the tumor cell cycle [36]. Notably, parainflammation, a low-grade form of inflammation, is widely prevalent in human cancer and relies mainly on macrophages in tissue [37, 38]. Although some highly expressed immune promotion-related cells, functions and pathways were found in the high-risk group, considering that our model is used as a whole to evaluate the relationship between the two groups and immune infiltration, the difference in the prognosis of the two groups should be the result of their internal interaction rather than attributed to a single factor alone.

In view of the above studies, we defined a prognostic model of 5 ferroptosis-associated genes that were independently associated with OS in both the derivation and validation cohorts, although this study has some limitations, such as a small sample size. Functional analysis and immune cell infiltration analysis confirmed that there was a certain correlation between our model and immune infiltration. The underlying mechanisms between ferroptosis-associated genes and tumor immune infiltration in LUSC are still poorly understood; nevertheless, this study provides new ideas to explore the mechanism of the initiation and progression of LUSC from the perspective of ferroptosis and immune cell infiltration.

## 5. Conclusions

In summary, our 5 ferroptosis-associated gene model can provide an evaluation reference for survival outcomes for patients with LUSC. This study indicates that ferroptosis has a certain relationship with immune infiltration in LUSC, and the infiltrating immune cells distinguished by this model in the high- and low-risk groups, such as M2 macrophages and immune-related pathways, were significantly related to the model, such as leukocyte transendothelial migration. Based on this, we speculate that this model may alter the expression of M2 macrophages by regulating leukocyte transendothelial migration, thereby regulating the immune microenvironment of LUSC and affecting the prognosis of patients. This study can provide a new understanding of ferroptosis in LUSC development and progression. Given that our results are based on RNA-seq technology, further research is needed to explore the prognostic value of this model.

## Supporting information

S1 Fig The expression of 5 genge. The expression of ALOX5(A), DPP4(B) are significantly lower in LUSC tissues than in 46 paired noncancerous adjacent tissues and the expression of FADS2(C), NOX1(D), and PHKG2(E) are opposite (all adjusted P<0.05).
(TIF)

S2 Fig Kaplan-Meier curves for the OS of patients in the high-risk group and low-risk group of 5 ferroptosis-related genes. The median score was used to divide patients into high expression and low expression groups. P < 0.05 means the difference is significant.
(TIF)

S3 Fig Construction of a 5-gene signature model in the TCGA cohort. A. LASSO coefficient profiles of the expression of 5 candidate genes. B. Selection of the penalty parameter (λ) in the LASSO model via 10-fold cross-validation. The dotted vertical lines are plotted at the optimal values following the minimum criteria (left) and “one standard error” criteria (right).
(TIF)

S4 Fig The abundance distribution of specific immune cells in different risk groups in the TCGA cohort. (TIF)

S5 Fig The significant relationship with the prognosis in breast cancer in the TCGA cohort. (TIF)

S6 Fig The significant relationship with the prognosis in hepatocellular carcinoma in the TCGA cohort. (TIF)

S1 Table Ferrotosis-related genes. (XLS)

S2 Table The annotated gene set file used in ssGSEA. (XLS)
