# Supplementary figures and images for: A survival model for prognostic prediction based on ferroptosis-associated genes and the association with immune infiltration in lung squamous cell carcinoma
Source: PLoS One. 2023 Mar 16;18(3):e0282888. doi: 10.1371/journal.pone.0282888 (PMC10019706; doi:10.1371/journal.pone.0282888)

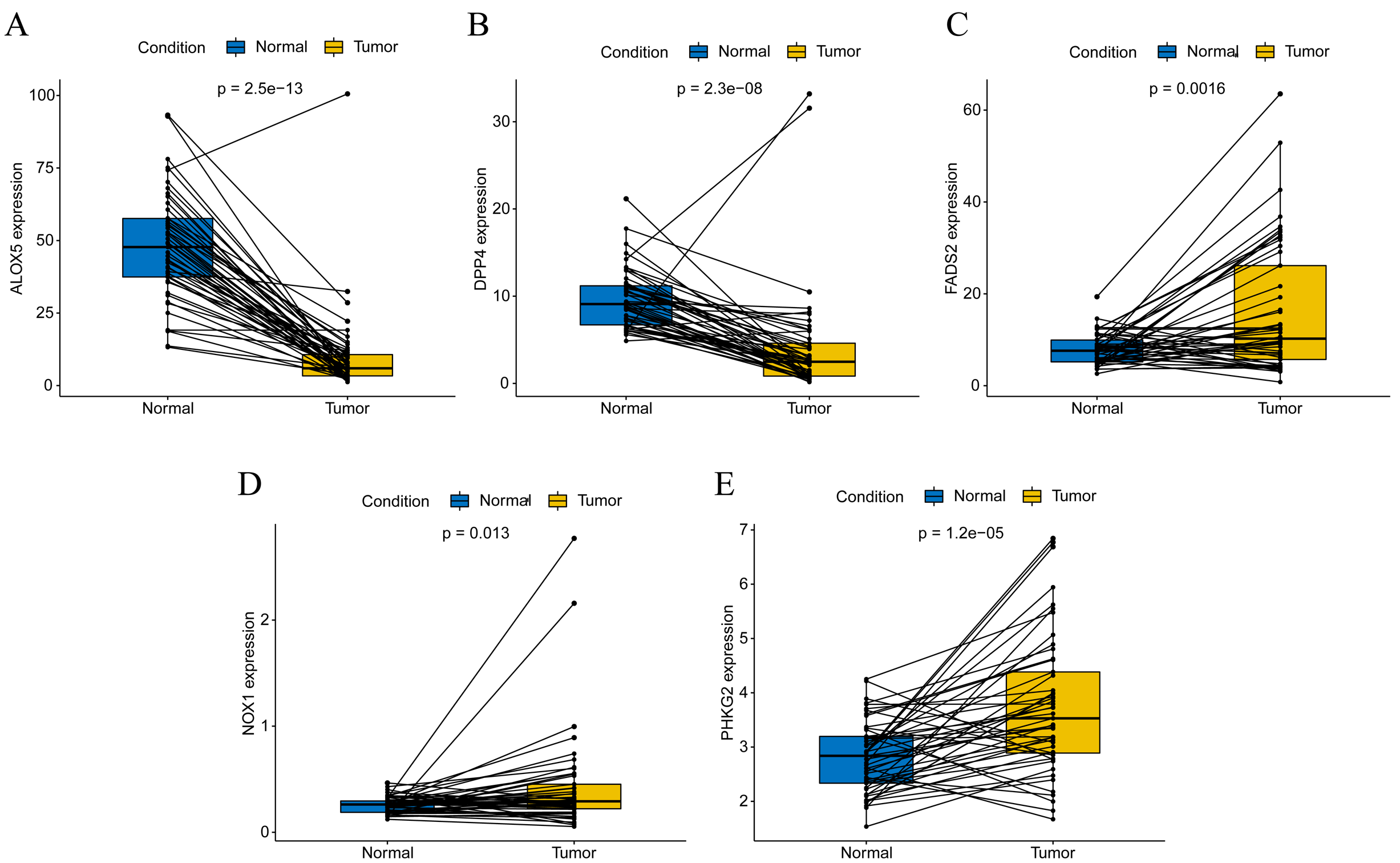

Supplement: S1 Fig — The expression of ALOX5(A), DPP4(B) are significantly lower in LUSC tissues than in 46 paired noncancerous adjacent tissues and the expression of FADS2(C), NOX1(D), and PHKG2(E) are opposite (all adjusted P<0.05). (TIF) [file pone.0282888.s001.tif]

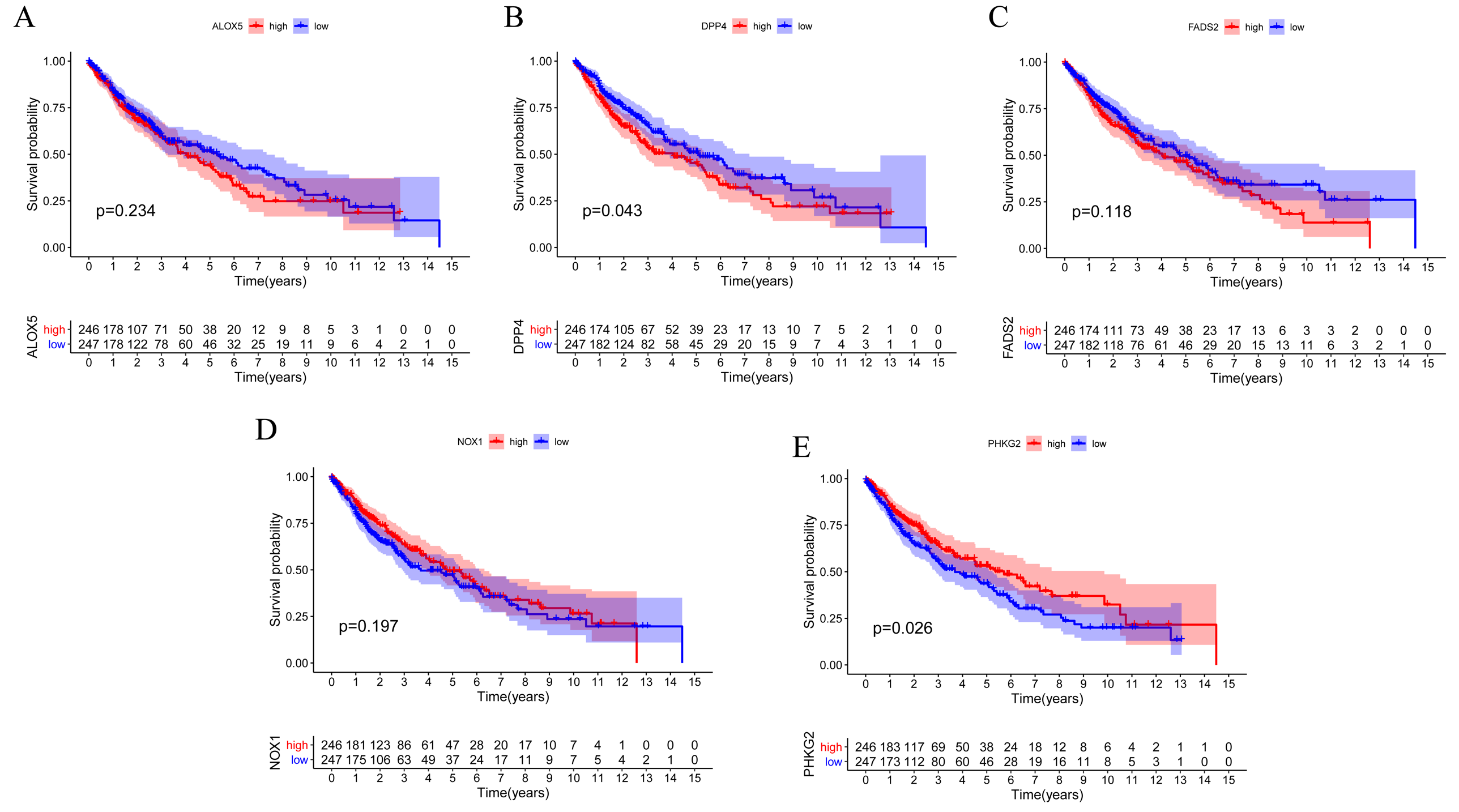

Supplement: S2 Fig — The median score was used to divide patients into high expression and low expression groups. P < 0.05 means the difference is significant. (TIF) [file pone.0282888.s002.tif]

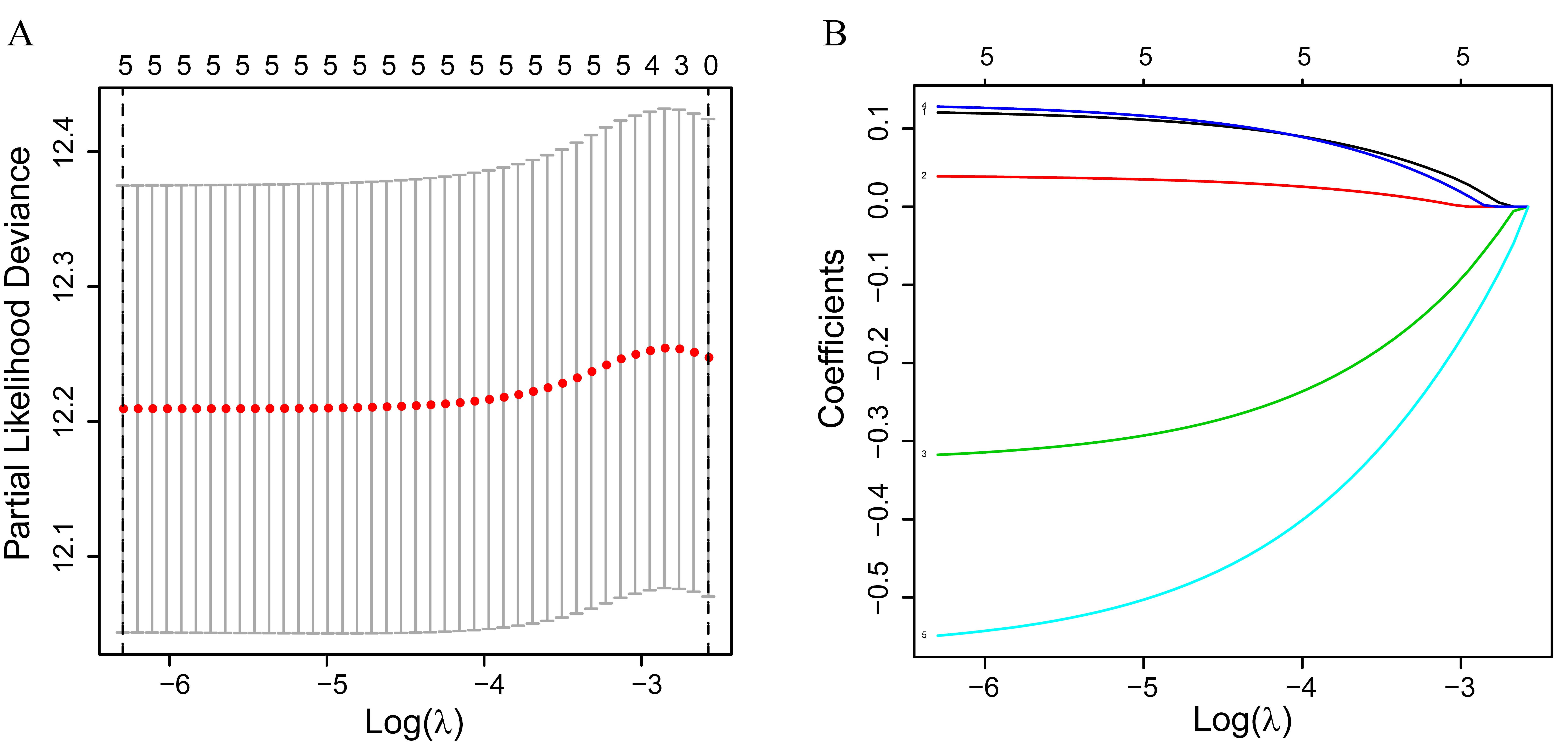

Supplement: S3 Fig — A. LASSO coefficient profiles of the expression of 5 candidate genes. B. Selection of the penalty parameter (λ) in the LASSO model via 10-fold cross-validation. The dotted vertical lines are plotted at the optimal values following the minimum criteria (left) and “one standard error” criteria (right). (TIF) [file pone.0282888.s003.tif]

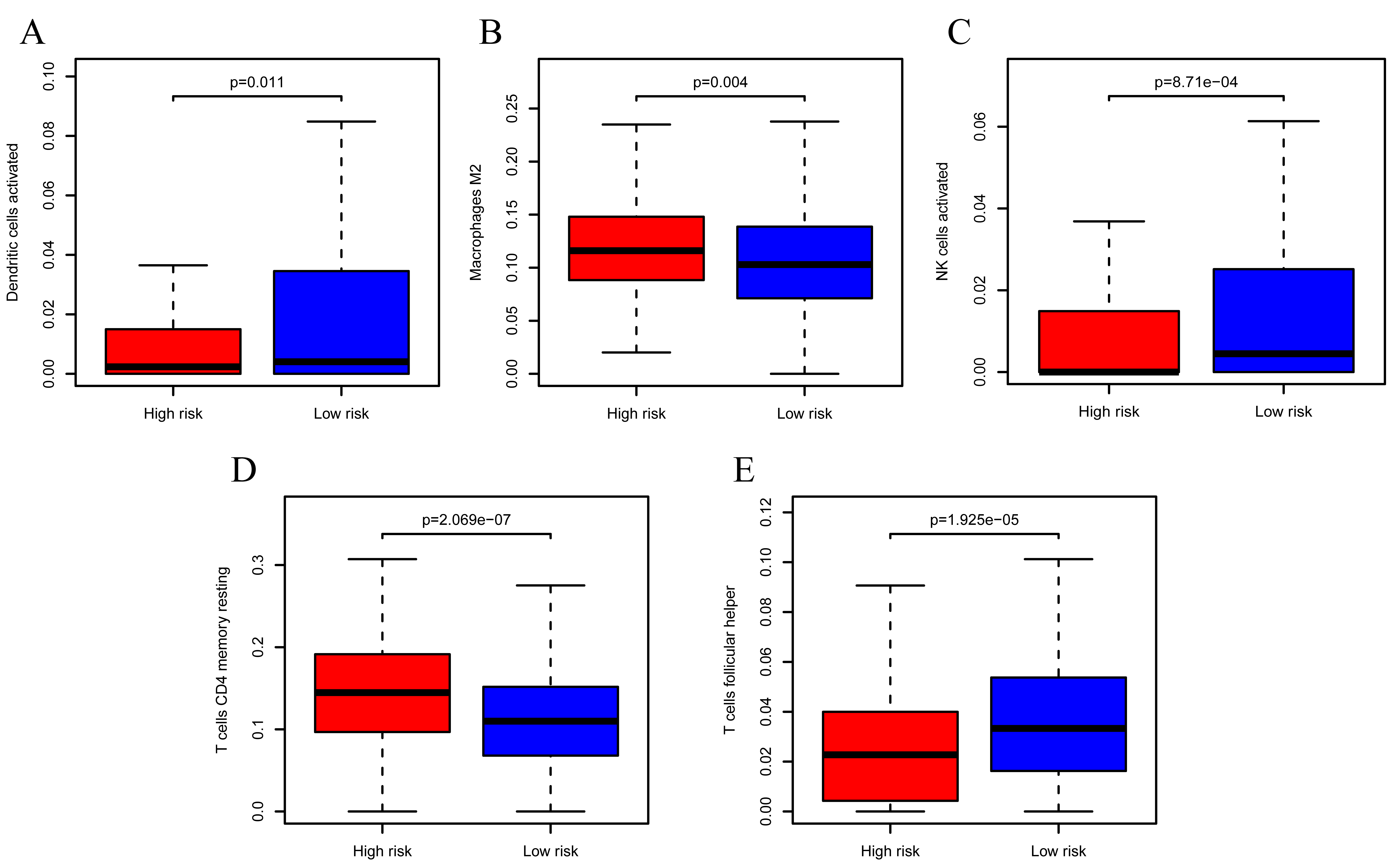

Supplement: S4 Fig — (TIF) [file pone.0282888.s004.tif]

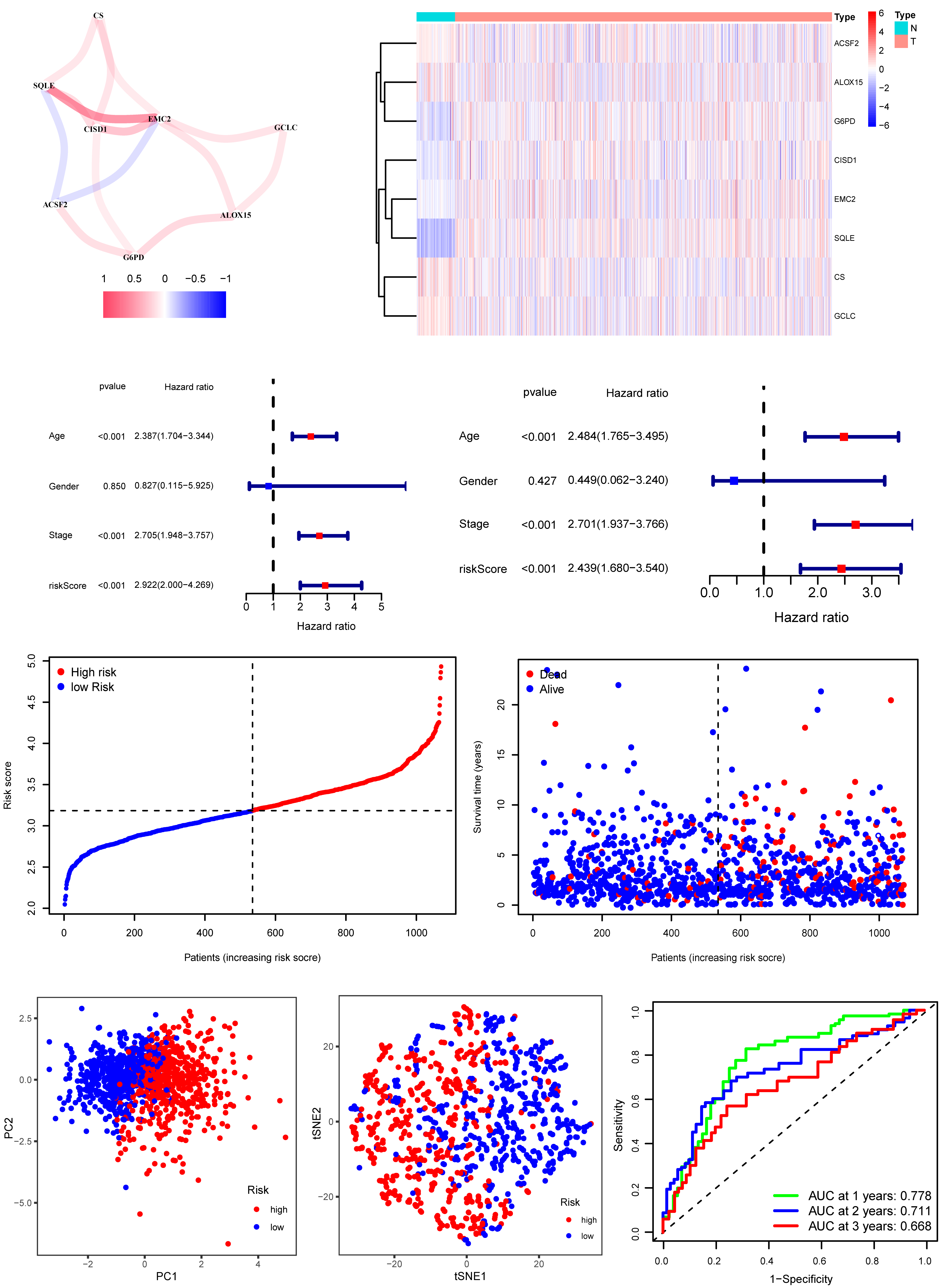

Supplement: S5 Fig — (TIF) [file pone.0282888.s005.tif]

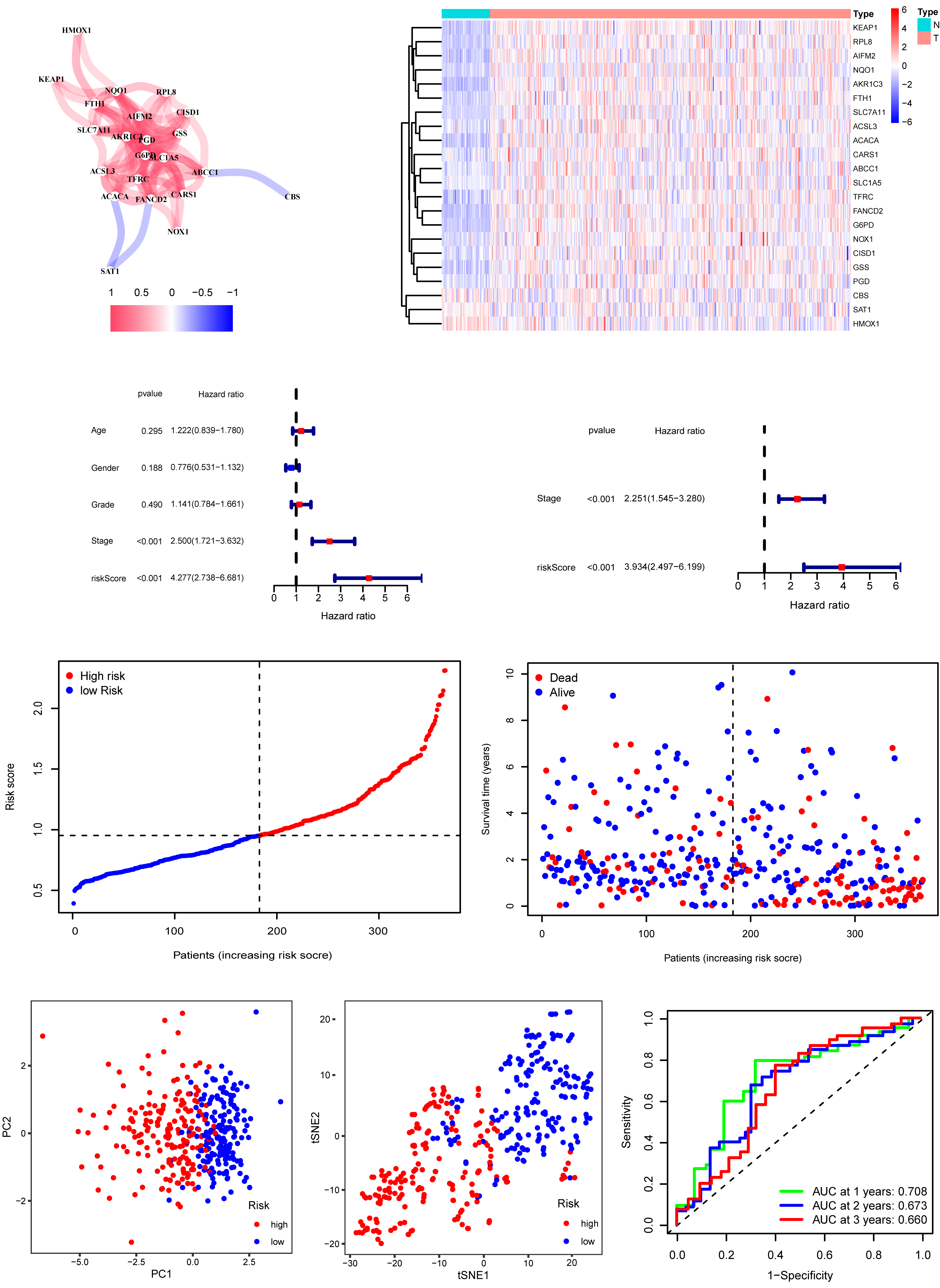

Supplement: S6 Fig — (TIF) [file pone.0282888.s006.tif]
